# Construction of the circRNA-miRNA-mRNA Regulatory Network of an Abdominal Aortic Aneurysm to Explore Its Potential Pathogenesis

**DOI:** 10.1155/2021/9916881

**Published:** 2021-11-05

**Authors:** Hao Zhang, Ce Bian, Simei Tu, Fanxing Yin, Panpan Guo, Jian Zhang, Yihao Wu, Yuhan Yin, Jiahui Guo, Yanshuo Han

**Affiliations:** ^1^School of Life and Pharmaceutical Sciences, Dalian University of Technology, Dalian, China; ^2^Department of Cardiovascular Surgery, The General Hospital of the PLA Rocket Force, Beijing Normal University, Beijing, China; ^3^Department for Vascular Surgery, First Hospital of China Medical University, Shenyang, China

## Abstract

**Background:**

Abdominal aortic aneurysm (AAA) is a progressive cardiovascular disease, which is a permanent and localized dilatation of the abdominal aorta with potentially fatal consequence of aortic rupture. Dysregulation of circRNAs is correlated with the development of various pathological events in cardiovascular diseases. However, the function of circRNAs in abdominal aortic aneurysm (AAA) is unknown and remains to be explored. This study is aimed at determining the regulatory mechanisms of circRNAs in AAAs. This study was aimed at exploring the underlying molecular mechanisms of abdominal aortic aneurysms based on the competing endogenous RNA (ceRNA) regulatory hypothesis of circRNA, miRNA, and mRNA.

**Methods:**

The expression profiles of circRNAs (GSE144431), miRNAs (GSE62179), and mRNAs (GSE7084, GSE57691, and GSE47472) in human tissue sample from the aneurysm group and normal group were obtained from the Gene Expression Omnibus database, respectively. The circRNA-miRNA-mRNA network was constructed by using Cytoscape 3.7.2 software; then, the protein-protein interaction (PPI) network was constructed by using the STRING database, and the hub genes were identified by using the cytoHubba plug-in. The circRNA-miRNA-hub gene regulatory subnetwork was formed to understand the regulatory axis of hub genes in AAAs.

**Results:**

The present study identified 40 differentially expressed circRNAs (DECs) in the GSE144431, 90 differentially expressed miRNAs (DEmiRs) in the GSE62179, and 168 differentially expressed mRNAs (DEGs) with the same direction regulation (130 downregulated and 38 upregulated) in the GSE7084, GSE57691, and GSE47472 datasets identified regarding AAAs. The miRNA response elements (MREs) of three DECs were then predicted. Four overlapping miRNAs were obtained by intersecting the predicted miRNA and DEmiRs. Then, 17 overlapping mRNAs were obtained by intersecting the predicted target mRNAs of 4 miRNAs with 168 DEGs. Furthermore, the circRNA-miRNA-mRNA network was constructed through 3 circRNAs, 4 miRNAs, and 17 mRNAs, and three hub genes (SOD2, CCR7, and PGRMC1) were identified. Simultaneously, functional enrichment and pathway analysis were performed within genes in the circRNA-miRNA-mRNA network. Three of them (SOD2, CCR7, and PGRMC1) were suggested to be crucial based on functional enrichment, protein-protein interaction, and ceRNA network analysis. Furthermore, the expression of SOD2 and CCR7 may be regulated by hsa_circ_0011449/hsa_circ_0081968/hsa-let-7f-5p; the expression of PGRMC1 may be regulated by hsa_circ_0011449/hsa_circ_0081968-hsa-let-7f-5p/hsa-let-7e-5p.

**Conclusion:**

In conclusion, the ceRNA interaction axis we identified may be an important target for the treatment of abdominal aortic aneurysms. This study provided further understanding of the potential pathogenesis from the perspective of the circRNA-related competitive endogenous RNA network in AAAs.

## 1. Introduction

Abdominal aortic aneurysm (AAA) is one of the cardiovascular diseases mainly distributed in Western countries and Asia, which mostly occurs in elderly men [1–3]. Local permanent dilation and weakening of the abdominal aorta are important characteristics of AAA [4]. Typically, degeneration of the abdominal aorta is a manifestation of a systemic process characterized by inflammation, apoptosis of smooth muscle cells, and destruction of elastin and collagen in the media and adventitia [5, 6]. More specifically, it would arise three clinical symptoms, asymptomatic and ruptured signs, and the characteristics of permanent expansion [7]. Generally, the mortality rate of AAA is up to 85%, which is inseparable from the severe bleeding and unpredictability caused by its rupture, and there is no effective treatment at present [8]. Nevertheless, AAA mortality data based on 1990-2010 from the Global Burden of Disease Study showed that the mortality of AAA decreased by 12.7% [9], and 43 percent of AAAs patients who were not included in the AAA screening criteria died, of which 9% were men and 34% were women [10]. Most of AAA patients were associated with asymptomatic AAA; therefore, the majority of AAA are identified incidentally during an examination for another unrelated pathology [11]. Therefore, the method to prevent the formation of aneurysms and the slow progress of aneurysms has become a growing public concern [12, 13].

Recently, several evidences have demonstrated that noncoding RNAs, such as long noncoding RNA (lncRNA), circular RNA (circRNA), and microRNA (miRNA), play an important role in the development of cardiovascular diseases [14–16]. CircRNA is spliced by exons at specific splicing sites to form a circular closed structure [17]. CircRNAs act as a competitive endogenous RNA (ceRNA) and compete with miRNA through its miRNA response element. miRNAs negatively regulate mRNA expression of protein-coding genes by binding to complementary sequences [18, 19]. Therefore, the circRNA-miRNA-mRNA interaction may be a significant mechanism in the occurrence and development of AAAs. According to our previous study, posttranscriptional modifications play important roles in modulating the functions of RNA involved in abdominal aorta aneurysm [20]. Yue et al. revealed the role of circCBFB/miR-28-5p/GRIA4/LYPD3 in the apoptosis of vascular smooth muscle cells (VSMCs), which conduced to the generation of new thoughts in AAA management [21]. Recently, Yang et al. investigated the role of circRNA CCDC66 in the pathogenesis of AAAs and determine its targeted miRNA and mRNA. The results show that CCDC66 will affect the proliferation of VSMCs through miR-342-3p/CCDC6 through circRNA CCDC66. They suggested that the overexpression of CCDC66 would induce the penetration of AAAs through the circCCDC66/miR-342-3p/CCDC66 pathway [22]. Meanwhile, Zhao et al. demonstrated that the pathogenesis of AAAs can be regulated by the CDR1as/miR-7/CKAP4 axis and affect the proliferation and apoptosis of primary vascular smooth muscle cells [23]. In summary, the circRNA-miRNA-mRNA network might play an important role in the pathogenesis of AAA, and circRNA has different targets and functions in different tissue cells.

Recently, Tian et al. performed microarray sequencing on human and rat abdominal aortic aneurysmal tissue and determined the differentially expressed lncRNA, miRNA, and mRNA by analyzing the public dataset and constructed the ceRNA network of lncRNA [24]. However, the specific targets and mechanisms of circRNA in AAAs have not been reported. Meanwhile, there are few reports on the ceRNA regulation mechanism of circRNA-miRNA-mRNA related to AAAs and on the interaction between ncRNA.

**Figure 1:**
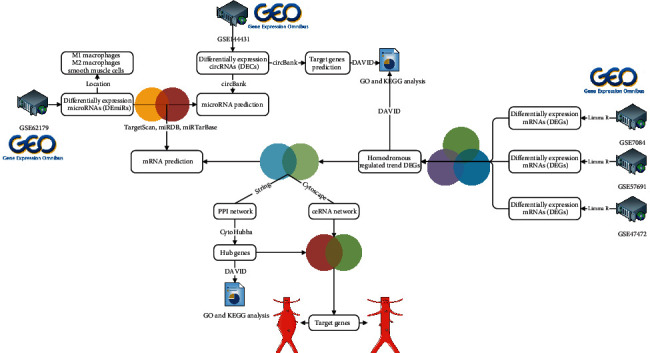
Flow diagram of data processing. The difference expression of three datasets (circRNA dataset, miRNA dataset, and mRNA dataset) was analyzed, and then, the intersection was selected; construction of the ceRNA network, protein-protein interaction network and functional enrichment analysis, and finally determination of the key genes.

In this study, the microarray data of circRNA, miRNA, and mRNA were downloaded from the Gene Expression Comprehensive (GEO) database, and RStudio was used to perform differential expression analysis of the microarray data to obtain differentially expressed circRNAs (DECs), differentially expressed miRNAs (DEmiRs), and differentially expressed genes (DEGs). Cytoscape 3.7.2 constructs the circRNA-miRNA-mRNA network, and the STRING (search tool for interactive gene retrieval) online analysis tool constructs the PPI network. This study is aimed at exploring novel circRNAs and their mechanisms in human tissues of AAA patients. The purpose of this study is to further screen the ceRNA axis of the key circRNA-miRNA-mRNA in AAAs with microarray data collected from public databases and bioinformatics methods. Besides, the findings of this study promoted the understanding of the molecular mechanism of AAAs, which provided a new direction for targeted therapy of AAAs. The circRNA-miRNA-mRNA regulatory subnetwork was formed to understand the regulatory axis of hub genes in AAA. Finally, Gene Ontology (GO) and Kyoto Encyclopedia of Genes and Genomes (KEGG) are introduced to reveal the potential impact of key genes in the development of AAA. [25]. The flowchart is shown in Figure 1.

## 2. Materials and Methods

### 2.1. Data Extraction

Microarray data of AAAs were downloaded from the National Center for Biotechnology Information (NCBI) GEO database (https://www.ncbi.nlm.nih.gov/geo/), with keywords searched such as “Abdominal aortic aneurysm” (AAA) and “Noncoding RNA” (circRNA, miRNA, and mRNA). During the retrieving process, “Homo sapiens” was set as the filtering condition; as a result, a total of five datasets were included in this study, namely, GSE144431, GSE62179, GSE7084, GSE47472, and GSE57691. As for circRNA expression data (GSE144431 dataset), the type of data was ncRNA profiling by array and the data of the microarray platform was 074301 Arraystar Human ncRNA microarray V2 (platform: GPL21825). In terms of the GSE62179 dataset, the type of data was miRNA profiling by array and the data of the microarray platform was Agilent-021827 Human miRNA Microarray G4470C (Feature Number version) (platform: GPL14767). Similarly, as for the three datasets (GSE7084, GSE47472, and GSE57691), the type of data was expression profiling by array and the data of the microarray platform was HG-U133_Plus_2 Affymetrix Human Genome U133 Plus 2.0 Array (platform: GPL570), Illumina HumanHT-12 V4.0 expression beadchip (platform: GPL10558), and Illumina HumanHT-12 V4.0 expression beadchip (platform: GPL10558), respectively. Each dataset was composed of AAA samples and healthy aorta samples. Series matrix files for differential expression analysis and other function analysis were downloaded from the Gene Expression Omnibus repository database. The box plot of datasets is shown in Supplementary Figure 1.

### 2.2. Differential Expression Analysis

GEO2R is a GEO database online analysis tool that can directly perform differential expression analysis. We directly exported the analysis results to make differential analysis. GEO2R and R package were employed to identify the differential expression of genes, and the corrected data matrix of each dataset was downloaded directly from the GEO database, with the differentially expressed gene (DEG) analysis method using the “Linear Models for Microarray Data (limma)” R package for the datasets. The annotation information of DEGs, differentially expressed circRNAs (DECs), and differentially expressed miRNAs (DEmiRs) of the three datasets were obtained with the GEO2R online software. The selection criteria for DECs included ∣log2FC (fold change)  | >1, and *P* value < 0.1 was considered to indicate a statistically significant difference. The selection criteria for DEGs included ∣log2FC (fold change) | >2, and *P* value < 0.05 was considered to indicate a statistically significant difference. There was statistical significance for the selection of this threshold, and those genes that were up- and downregulated can also be selected for the subsequent analysis. The R software package was also adopted to draw a heat map of differentially expressed circRNAs and differentially expressed mRNAs.

In this study, these differentially expressed genes in the three mRNA databases were divided into the upregulated and downregulated groups. Subsequently, the Venn diagram was employed to obtain mRNAs that were regulated in the same regulated pattern, and Venny (version 2.1.0) (https://bioinfogp.cnb.csic.es/tools/venny/), the online mapping software, was utilized to create the Venn diagram. The R software package was used for functional enrichment and pathway analysis of differential genes. Due to the different types of miRNA data and different miRNAs in AAA groups were extracted compared with normal groups with the aim of exploring DEmiRs in different aneurysm cells, and the network was constructed at the end.

### 2.3. Protein-Protein Interaction Network Analysis of DEGs

STRING protein interaction prediction (version110.b) (STRING: functional protein association networks (string-db.org)) was used to search for known protein interactions and predict protein interactions. The intersections of the differentially expressed mRNA target genes were taken in a consistent manner from three mRNA datasets and were imported into the database. After the points without interaction were hidden, the data were imported into Cytoscape (version 3.7.2) to visualize the protein-protein interaction (PPI) network.

### 2.4. Prediction of circRNA-miRNA Pairs

The interaction relationships between circRNAs and miRNA were predicted with circBank (http://www.circbank.cn/help.html) [26]. circBank was employed to predict circRNA. Overlapping miRNAs were obtained by intersecting the predicted circRNAs and DEmiRs.

### 2.5. Prediction of Target Genes of miRNAs

The miRNA-mRNA interactions were based on the following three miRNA target prediction databases: miRTarBase (https://maayanlab.cloud/Harmonizome/resource/MiRTarBase), TargetScan (http://www.targetscan.org/vert_72/), and miRDB (http://mirdb.org/). Perl (version 5.32.0) was adopted to analyze DEmiRs and the targeted mRNAs. Overlapping mRNAs were obtained by intersecting the predicted mRNAs and DEmiRs.

### 2.6. CircRNA-miRNA-mRNA ceRNA Network Construction

In this study, the intersection of the three databases was selected as the results. The ceRNA network was constructed based on the commonly interactive differentially expressed mRNAs with differentially expressed miRNAs and differentially expressed circRNAs. The circRNA-miRNA-mRNA ceRNA network was visualized with Cytoscape (version 3.7.2) (https://cytoscape.org/).

### 2.7. Functional Enrichment and Pathway Analysis

To explore the main functions and pathways of DEGs derived from differentially expressed circRNA target genes, differentially expressed mRNA target genes in a consistent manner, and target genes of the circRNA network, the Database for Annotation, Visualization and Integrated Discovery online tool (version 6.8) (DAVID: https://david.ncifcrf.gov/) was introduced for the Gene Ontology (GO) and Kyoto Encyclopedia of Genes and Genomes (KEGG) pathway enrichment analyses. The gene lists were downloaded for the biological function information.

### 2.8. Differential Expression Analysis of Key Genes

The plug-in cytoHubba in the Cytoscape software is visualization software that obtains the dense relationship through the degree, betweenness centrality, and closeness centrality algorithms. To obtain the key genes in the PPI network, we identified the hub genes in the PPI network through cytoHubba. Key genes were obtained through the intersection of Venn diagram PPI network hub genes and ceRNA network genes. To accurately explore the differential expression of key genes in the three datasets, we used the R language software package to draw box plots of key genes.

## 3. Results

### 3.1. Differential Expression Analysis

**Figure 2:**
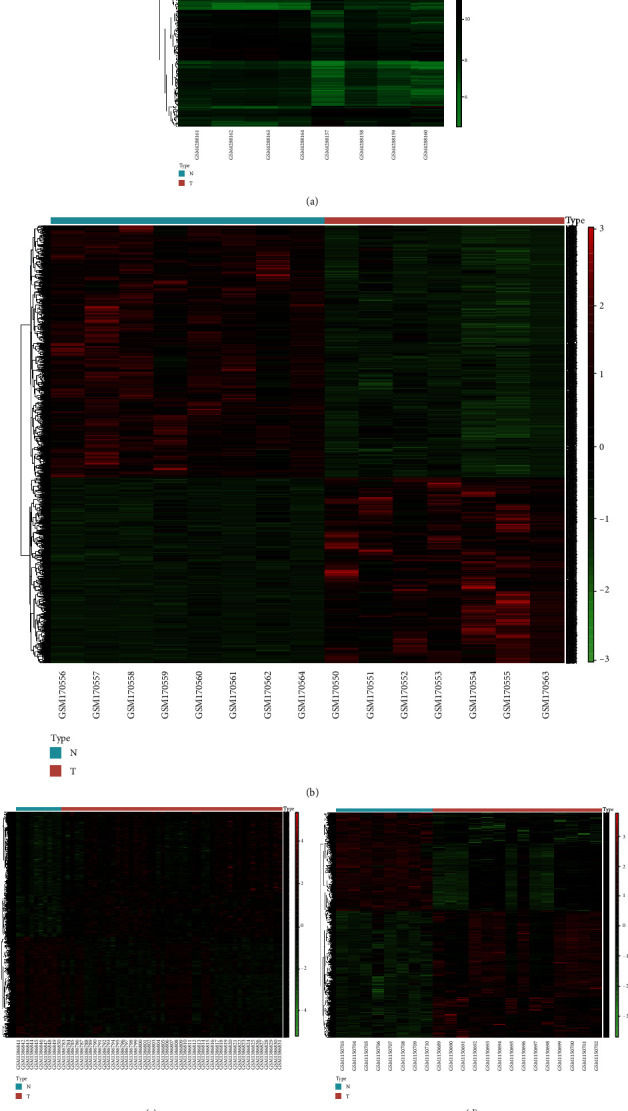
Hierarchical clustering and heat map analysis of differentially expressed circRNAs (a) and mRNAs (b–d). (a) GSE144431, circRNA; (b) GSE7084, mRNA; (c) GSE57691, mRNA; (d) GSE47472, mRNA. The green color indicates low expression, the red indicates high expression. Column: an aortic sample. Differentially expressed circRNA molecules were screened under the cut-off criteria ∣log2FC | >1 and the *P* value (*P* < 0.1). Differentially expressed mRNA molecules were screened under the cut-off criteria ∣log2FC | >2 and the *P* value (*P* < 0.05).

**Figure 3:**
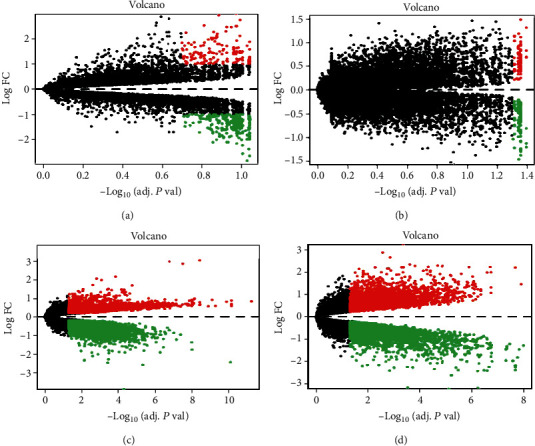
Volcano plots of differentially expressed circRNAs (a) and mRNAs (b–d). (a) GSE144431, circRNA; (b) GSE7084, mRNA; (c) GSE57691, mRNA; (d) GSE47472, mRNA. Green spots: downexpressed RNA molecules; red spots: upexpressed RNA molecules. Differentially expressed circRNA molecules were screened under the cut-off criteria ∣log2FC | >1 and the *P* value (*P* < 0.1). Differentially expressed mRNA molecules were screened under the cut-off criteria ∣log2FC | >2 and the *P* value (*P* < 0.05).

According to the threshold criteria (*P* < 0.1 and ∣log2FC | >1), the GSE144431 datasets contained circRNA data, and a total of 382 differentially expressed circRNAs were visualized by hierarchical clustering heat map analysis (Figure 2(a)) and volcano plot analysis (Figure 3(a)), with a view to endowing the selection results with much significance. A total of 40 samples ranking for the top 40 with the most significant differences were selected, including 20 upregulated DECs and 20 downregulated DECs (Table 1).

**Figure 4:**
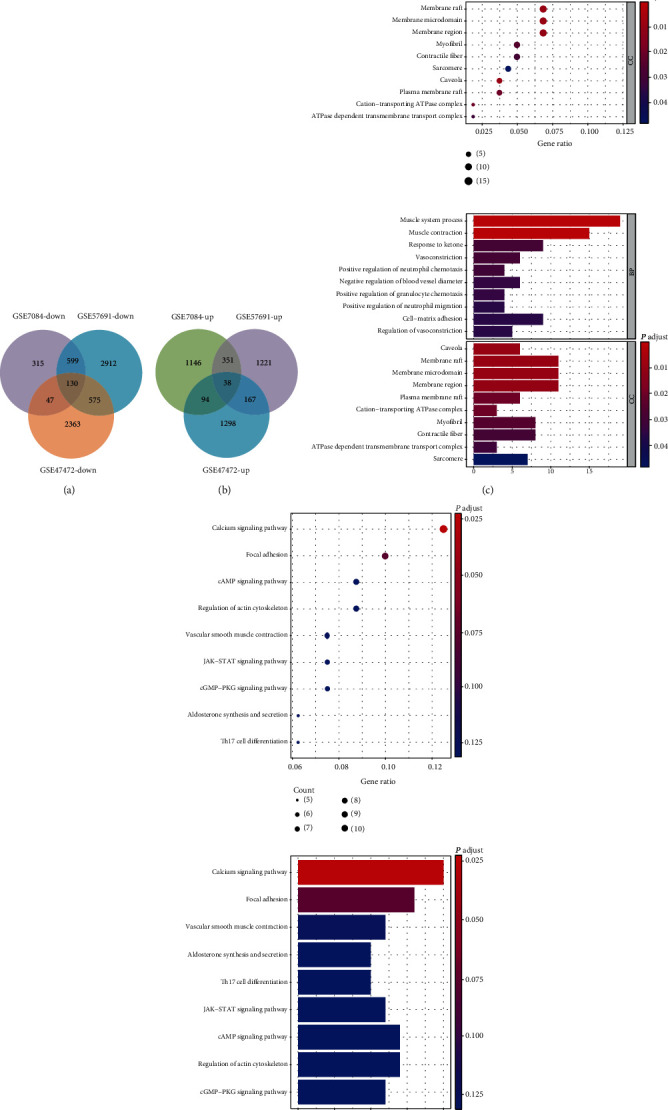
Venn diagram analysis and GO/KEGG analysis of 168 DEG coregulation modes in the three datasets: (a) intersection of downregulated genes (GSE7084, GSE57691, and GSE47472); (b) intersection of upregulated genes (GSE7084, GSE57691, and GSE47472); (c) bubble chart and barplot of 168 DEGs by GO analysis; (d) bubble chart and barplot of 168 DEGs by KEGG analysis.

The mRNA datasets of GSE7084, GSE57691, and GSE47472 contained mRNA microarray data. All differentially expressed mRNAs in GSE7084, GSE57691, and GSE47472 were selected for hierarchical clustering heat map analysis and visualization (Figures 2(b)–2(d)); volcano plots are shown in Figures 3(b)–3(d). Simultaneously, the common mRNAs were extracted from three differentially expressed datasets for the first time, and a total of 215 common DEGs were obtained at the end. Among all results, the top 20 items with the most significant *P* values were selected and included in Table 1. As for 215 common DEGs, it could be inferred from that Protein Phosphatase 1 Catalytic Subunit Beta (PPP1CB) and Transmembrane Protein 47 (TMEM47) were upregulated in the GSE47472 and downregulated in the GSE7084 and GSE57691. Similarly, G Protein-Coupled Receptor Class C Group 5 Member C (GPRC5C) was upregulated in the GSE57691 and downregulated in the GSE7084 and GSE47472 (Table 1). Therefore, the adverse regulated DEG results could not be regarded as the content of further analysis. In order to make the research results obtained more accurate, those DEGs with a different regulated trend in the three microarray databases were excluded. Subsequently, the DEGs with the same regulated trend in the three databases were intersected and 168 DEGs were selected. Among them, 130 DEGs were downregulated (Figure 4(a)) and 38 DEGs upregulated (Figure 4(b)). The GO analysis of 168 DEGs involves the biological process and cellular component, and biological process included vasoconstriction, negative regulation of blood vessel diameter, and cell-matrix adhesion (Figure 4(c)). The KEGG analysis of 168 differentially expressed genes included the calcium signaling pathway, vascular smooth muscle contraction, and focal adhesion (Figure 4(d)); 47 common DEGs of different regulation methods in the three datasets are listed in Supplement Table 1.

The GSE62179 dataset contained miRNA data, and there were 90 differentially expressed miRNAs in the control group compared with the AAA groups of M1 macrophages, M2 macrophages, and smooth muscle cells (SMCs) (Table 2).

### 3.2. PPI Network for DEGs

**Figure 5:**
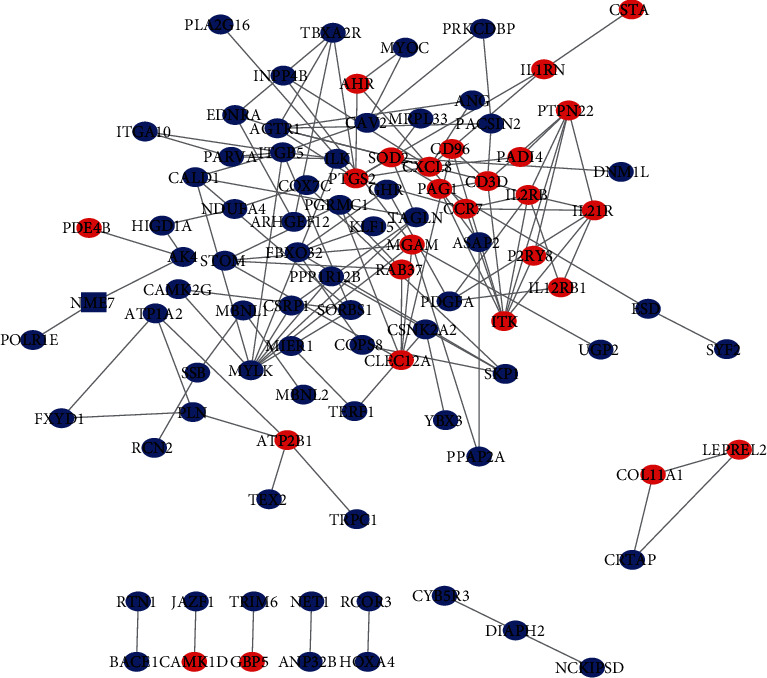
Protein and protein interaction network for DEG coregulation modes. Dots represent gene expression, and lines represent interaction relationships. Blue: downregulated; red: upregulated.

When the consistent differentially expressed mRNAs in the GSE7084, GSE47472, and GSE57691 were imported to the STRING website, it could be found that there were 131 interaction relationships for 168 DEGs, which were adopted to construct the PPI network. After the points that are not connected and interacted were hidden, the PPI network file was downloaded. In the present network, AHR could interact with MYOC, PTGS2, and CXCL8; CCR7 could interact with PRKCDBP, IL2RB, ITK, IL21R, PTGS2, and CXCL8; SOD2 could interact with CXCL8, DNM1L, ESD, MRPL33, PTGS2, and TAGLN (Figure 5). Furthermore, cytoHubba was used to identify hub genes with high accuracy according to the network topology. After the filtering condition was set according to the maximal clique centrality (Mcc), the determined top 30 hub genes could be obtained, including IL2RB, ITK, IL21R, MYLK, PTPN22, CXCL8, PTGS2, ITGB5, CD3D, AGTR1, CLEC12A, SORBS1, CCR7, STOM, CALD1, PDGFA, PGRMC1, RAB37, MGAM, SOD2 and etc, which may indicate potential regulatory networks and regulatory pathways.

### 3.3. Prediction of circRNA-miRNA Pairs

The circRNA-miRNA pairs corresponding to 40 DECs were predicted by using the Circular RNA Interactome online software (circBank), which is used for online prediction of miRNA bound to circRNA by combining with circBank. The 2230 miRNA interaction pairs combined with circRNA were predicted by using circBank; meanwhile, only the last five results of miRNA bound to circRNA were shown here (Supplementary Table 2). The predicted results were intersected with the differentially expressed miRNAs of the dataset, and 22 miRNAs could be obtained.

### 3.4. Prediction of Target Genes of miRNAs

Moreover, there were 957 mRNAs predicted to bind to 22 miRNAs by compilations of the miRTarBase database, TargetScan database, and miRDB database. The results obtained were intersected with 168 DEGs, and as a result, there were 17 potential mRNAs included.

### 3.5. Construction of the circRNA-miRNA-mRNA Network

**Figure 6:**
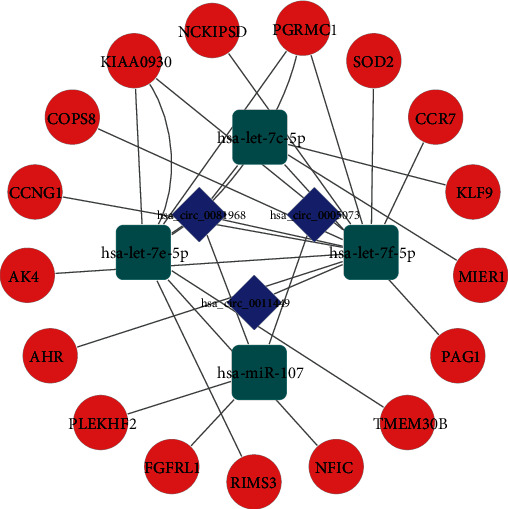
The ceRNA interaction network of circRNA-miRNA-mRNA. Round represents mRNAs, square shapes represent miRNAs, and triangles represent circRNAs. Connection represents interaction.

After the points where circRNA-miRNA-mRNA did not connect and interact were removed, circRNA-miRNA-mRNA regulatory networks were constructed. There were 3 DECs, 4 DEmiRs, and 17 DEGs in the ceRNA network (Figure 6), and KIAA0930 and PGRMC1 can be targeted by two miRNAs (hsa-let-7f-5p and hsa-let-7e-5p). These two DEmiRs could also interact with hsa_circ_001149 and hsa_circ_0081968, which would form the following ceRNA relationships, namely, hsa_circ_0081968/hsa_circ_0011449-hsa-let-7f-5p/hsa-let-7e-5p-KIAA0930/PGRMC1. Therefore, hsa_circ_005073/hsa_circ_0081968-hsa-miR-107-FGFRL1/PLEKHF2 and hsa_circ_0081968/hsa_circ_0011449-hsa-let-7f-5p-SOD2 ceRNA networks also could be obtained.

Eventually, the circRNA-miRNA-hub gene subnetwork with six regulatory modules, including the hsa_circ_0011449/hsa-let-7f-5p/SOD2 regulatory axis, hsa_circ_0081968/hsa-let-7f-5p/SOD2 regulatory axis, hsa_circ_0011449/hsa-let-7f-5p/CCR7 regulatory axis, hsa_circ_0081968/hsa-let-7f-5p/CCR7 regulatory axis, hsa_circ_0081968/hsa-let-7e-5p/PGRMC1 regulatory axis, and hsa_circ_0081968/hsa-let-7f-5p/PGRMC1 regulatory axis, was constructed to depict the relationship among circRNAs, miRNAs, and hub genes.

### 3.6. Functional Enrichment and Pathway Analysis

DECs included 20 differentially expressed circRNAs, while DEGs included 168 consistent differentially expressed mRNAs. GO and KEGG analyses were applied on DECs and DEGs, respectively. These DECs were analyzed through the three processes of Gene Ontology (GO) analysis, including biological processes (BP), cellular components (CC), and molecular functions (MF). KEGG analysis was applied to find the functional pathway of those genes, with the findings showing that DEGs in 20 differentially expressed circRNAs were enriched into eight GO biological process terms, e.g., nucleoplasm and actin cytoskeleton and 10 KEGG pathways such as the activation of Rac and Beta2 integrin cell surface interactions (Table 3).

Due to the fact that consistent overlapping DEGs were collected from three mRNA microarray datasets, they were divided into two parts for GO and KEGG analysis (the downregulation group and the upregulation group). In the downregulation group, there were 23 GO biological process terms and 5 KEGG pathways, e.g., positive regulation of cell migration (MYOC, NTRK3, PDGFA, ILK, LGR6, and MYLK), caveola assembly (CAV2 and PACSIN2), cGMP-PKG signaling pathway (EDNRA, PLN, AGTR1, ATP1A2, and MYLK), and vascular smooth muscle contraction (EDNRA, ARHGEF12, CALD1, AGTR1, PPP1R12B, and MYLK) (Table 4). In the upregulation group, there were 14 GO biological process terms and 3 KEGG pathways, e.g., T cell receptor signaling pathway (ITK, PDE4B, PTPN22, CD3, and PAG1), negative regulation of cell proliferation (CXCL8, RASSF5, SOD2, and PTGS2), cytokine-cytokine receptor interaction (CXCL8, IL2RB, IL21R, CCR7, and IL12RB1), and chemokine signaling pathway (ITK, CXCL8, and CCR7) (Supplementary Table 3).

To reveal the biological mechanism or functional pathway of the ceRNA network, DEGs in the ceRNA network were also subjected to GO/KEGG analysis, with the findings of functional enrichment showing that there were six GO biological process terms and two KEGG pathways, e.g., liver development (AK4 and SOD2), COP9 signalosome (NCKIPSD and COPS8), and transport vesicle (PLEKHF2 and FGFRL1) for GO analysis and peroxisome (SOD2) and FoxO signaling pathway (SOD2) for the KEGG pathway (Table 5).

### 3.7. Differential Expression Analysis of Key Genes

**Figure 7:**
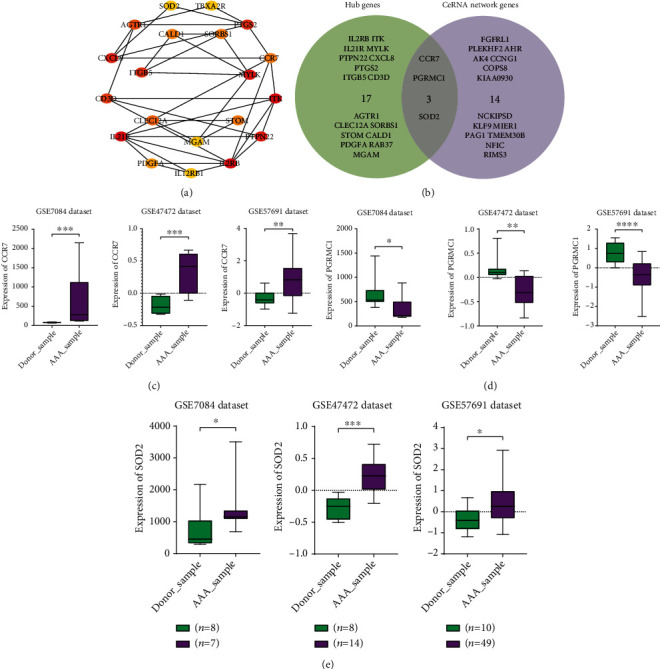
Differential expression analysis of hub genes from PPI network and ceRNA network genes. (a) Hub gene relationship networks from the PPI network. (b) Venn diagram analysis of hub genes from PPI network and ceRNA network genes. (c) Box plot of the differentially expressed gene CCR7 in the three datasets. (d) Box plot of the differentially expressed gene PGRMC1 in the three datasets. (e) Box plot of the differentially expressed gene SOD2 in the three datasets. Green: normal samples; purple: aneurysm samples; *n*: number of samples.

The top 20 genes obtained by the degree, betweenness centrality, and closeness centrality algorithms in cytoHubba are listed in Figure 7(a); overlapping genes of the PPI network and ceRNA network were selected as hub genes SOD2, CCR7, and PGRMC1 (Figure 7(b)); the differential expression analysis of the hub genes in the three datasets is shown in Figures 7(c)–7(e). Compared with the control group, SOD2 was upregulated in the three datasets, CCR7 was upregulated in the three datasets, and PGRMC1 was downregulated in the three datasets.

## 4. Discussion

In this study on AAAs, it has been confirmed that noncoding RNAs exert significant impacts on the pathogenesis of the disease [27, 28]. CircRNA is a stable noncoding RNA that has long been neglected by transcriptomics due to the lack of a 5′ cap and a 3′ polyadenylated tail. As recent studies, there are many endogenous circRNAs in mammalian cells, some of which show high abundances and evolutionary conservation. It has been initially indicated that circRNAs could mediate miRNA functions (e.g., via sponging) and control important events in transcription (e.g., RNA folding and endonuclease protection) [19]. In addition, it has been demonstrated in current evidence that circRNAs contain multiple microRNA response elements (MREs) that can bind to miRNAs, commonly called “miRNA sponges,” which could relieve the targeted inhibition of downstream mRNAs by miRNAs [29–31]. In this study, the underlying molecular mechanisms of AAAs have been investigated based on ceRNA regulatory hypothesis of circRNAs, miRNA, and mRNA. The expression profiles of circRNAs, miRNAs, and mRNAs in human tissue samples from the aneurysm group and the normal group have been identified.

In the three RNA (circRNA, miRNA, and mRNA) microarray datasets, 17 DEGs have been identified in the circRNA-miRNA-mRNA network, and they form a ceRNA regulatory network. Hub genes, as key genes, could exert decisive impacts on the biological processes. The regulation of other genes in the relevant pathway would be frequently affected by hub genes. Therefore, it is of great significance to find the hub genes for potential targets or research hotspots. CytoHubba is effective software of the Cytoscape plug-in, which is used to identify hub genes with high accuracy according to the network topology. Within bioinformatics analysis, three hub genes (SOD2, CCR7, and PGRMC1) were identified.

Strauss et al. suggested that SOD2 may be related to the formation of abdominal aortic aneurysm through oxidation reaction [32]. However, there are few reports on the effect of SOD2 genes in AAAs. Superoxide dismutase (SOD) is a ubiquitous antioxidant enzyme that catalytically converts the superoxide radical to hydrogen peroxide (H_2_O_2_) [33, 34]. The copper/zinc- (SOD1) and manganese- (MnSOD, SOD2) requiring superoxide dismutases are important and major antioxidant enzymes located in the mitochondria and exert decisive impacts on the progression of tumors [35, 36]. For instance, epigenetic silencing of SOD2 in KAS 6/1 human multiple myeloma cells can increase cell proliferation [37]. The blocking of SOD2 expression would significantly inhibit TNF-*α*-induced cell proliferation in A549 and H1299 cells in vitro. Therefore, TNF-*α*-mediated lung inflammation can upregulate SOD2 expression in lung adenocarcinoma, and macrophages contribute to SOD2 upregulation by secreting TNF-*α* [38]. In cardiovascular disease, SOD2 stands at the forefront against mitochondrial ROS in vascular smooth muscle cells (VSMCs) via its preferential localization to the mitochondria, which conduces to the potential modification of the probability of vascular calcification [39] initiation or progression. Moreover, SOD2 augmentation may be a promising therapeutic strategy for the prevention of lesion formation in proliferative vascular diseases such as restenosis [40]. Madamanchi et al. suggested that SOD2 could regulate SMC quiescence by suppressing divergent mitogenic signaling pathways, and the dysregulation of these enzymes under pathophysiological conditions may induce SMC hyperplasia and hypertrophy [41]. The expression level of SOD2 will also have an influence on the migration of VSMCs and the formation of intima. Mechanically, Qu et al. suggested that peroxisome proliferator-activated receptor-gamma coactivator-1alpha (PGC-1alpha) might upregulate the expression of the mitochondrial antioxidant enzyme SOD2, which would inhibit VSMC migration and neointimal formation after vascular injury in rats [42]. On the contrary, it has been reported in other studies that the loss of heterozygous SOD2 would aggravate chronic intermittent hypoxia-induced lung inflammation and vascular remodeling through the mtROS-NLRP3 signaling pathway [43]. Finally, the increase in SOD2 expression in genotyped primary human umbilical vein endothelial cells (ECs) induced by fluid shear stress (FSS) would effectively stabilize their antiatherosclerotic phenotype [44]. Besides, it has also been found in this study that SOD2 may exert its impacts through hsa_circ_0011449/hsa_circ_0081968/hsa-let-7f-5p. To sum up, it has been indicated in those studies that the dysregulation of SOD2 can cause related arterial diseases or changes in the endothelial cell or smooth muscle cell function and phenotype. Therefore, the research on SOD2 and its functions may become a popular trend for the treatment of AAAs. Considering the important role that SOD2 may play in AAAs, some papers have been found to explain the effects of SOD2 on arteries.

At the same time, it is also the most significant among the differentially expressed genes in AAAs. hsa-let-7f-5p can form a regulatory network with many differentially expressed genes, for example, interaction with AHR (Aryl Hydrocarbon Receptor). Besides, Fibroblast Growth Factor Receptor-Like 1 (FGFRL1) and Pleckstrin Homology and FYVE Domain Containing 2 (PLEKHF2) may affect the transportation of intracellular substances to promote aneurysm formation, through the hsa_circ_0005073/hsa_circ_0081968/hsa-miR-107 regulated axis. There are relatively few studies on PLEKHF2 and FGFRL1 to indicate that they may exert significant impacts on the occurrence and development of AAAs. E. Shamsara and J. Shamsara have confirmed that the amplification of the PLEKHF2 gene is related to the decreased survival of patients with prostate cancer [45]. FGFRL1 would be significantly upregulated in patients with ovarian cancer, and high FGFRL1 expression is associated with a poor prognosis. The loss of FGFRL1 function significantly affects the proliferation, apoptosis, and migration of ovarian cancer cells in vitro and tumor growth in vivo [46]. For hsa-miR-107, Wang et al. have found that Integral Membrane Protein 2C (ITM2C) would be underexpressed and miR-107-5p overexpressed in acute aortic dissection tissues [47]. Meanwhile, the overexpression of miR-107-5p would promote cell proliferation and inhibit cell apoptosis in rat aortic smooth muscle cells (RASMCs).

CCR7 is a proinflammatory cytokine and is found in human atherosclerotic plaques [48]. It has been found that the expression of CCR7 is dramatically downregulated in human carotid atherosclerotic plaques [49]. Wan et al. have assessed the protein expression of CCR7 in AAAs through immunohistochemistry, with the findings showing that CCR7 is obviously upregulated in AAA compared with the control healthy aorta [50]. Recently, the homeostatic chemokines, CCL19 and CCL21 and their receptor CCR7, have been linked to atherogenesis. The current findings suggest that CCR7 is immunohistochemically localized to macrophages and vascular SMCs via carotid atherosclerosis and highlight that although CLL19 and CCL21 are signaling through CCR7, they may have different effects on macrophages and SMC [51]. In this study, it has also been found that the hsa_circ_0081968/hsa-let-7f-5p/CCR7 regulatory axis may be an important target for the treatment of AAAs. Cai et al. have also revealed that a lncRNA/miRNA/mRNA interaction network may be involved in the pathogenesis of pulmonary arterial hypertension (PAH) and suggested hsa-let-7e-5p and CCR7 can be regarded as potential biomarkers for PAH [52].

However, there are some limitations in this study. Firstly, although it has been shown that SOD2 can cause arterial-related diseases, there is no research to prove the relationship between abdominal aortic aneurysm and SOD2. Secondly, the way of action has been predicted based on the measured RNA network, which, however, has not been confirmed (dual-luciferase reporter gene analysis, gene overexpression, or gene knockout). Although several related genes have been screened out in this study for the first time, further in vitro clinical research and in vivo experiments shall be carried out to confirm its expression and functional mechanism in terms of AAAs.

At present, there are relatively few articles on the circRNA-miRNA-mRNA network regulating AAA, and most of them are ceRNA regulatory networks based on lncRNA. Based on Gene Expression Omnibus, we detected six dysregulations of circRNA-miRNA-mRNA axes in the AAA condition compared with healthy aortic condition. The novelty of this study is that the circRNA-miRNA-mRNA network has been constructed for the first time through the GEO database. However, given that these findings are only based on bioinformatics models, it is necessary to conduct a thorough study to verify the possible effects of these 6 axes in AAAs. Further studies are needed to elucidate the biological function of these circRNAs in the pathogenesis of AAA.

## Figures and Tables

**Table 1:** Crucial differentially expressed genes, circRNA, and mRNA between normal tissue and AAAs.

mRNA							circRNA					
	GSE7084		GSE47472		GSE57691		GSE144431					
Gene symbol	*P* value	LogFC	*P* value	LogFC	*P* value	LogFC	circRNA	LogFC	*P* value	circRNA	LogFC	*P* value
PTPN22	2.90*E* − 06	1.1005	3.30*E* − 05	1.4028	9.05*E* − 06	0.5580	hsa_circ_0005073	-2.6376	6.53*E* − 05	hsa_circ_0037128	1.0436	1.02*E* − 05
TBXA2R	6.58*E* − 06	-1.4859	4.23*E* − 08	-1.2298	5.44*E* − 08	-0.8940	hsa_circ_0006845	-1.7609	1.10*E* − 04	hsa_circ_0017972	1.0567	2.13*E* − 05
NCF4	7.51*E* − 06	2.0513	1.33*E* − 04	-0.8086	5.78*E* − 04	0.4700	hsa_circ_0044885	-0.7655	1.11*E* − 04	hsa_circ_0039557	0.9765	5.14*E* − 05
GPRC5C	9.36*E* − 06	-2.3531	1.58*E* − 04	-0.9018	2.97*E* − 03	0.3340	hsa_circ_0011449	-1.4614	1.11*E* − 04	hsa_circ_0062011	0.8934	9.86*E* − 05
PPP1CB	1.88*E* − 05	-1.1376	1.01*E* − 04	0.8658	4.16*E* − 06	-1.2800	hsa_circ_0082140	-1.1576	1.42*E* − 04	hsa_circ_0000690	1.0040	1.26*E* − 04
CRTAP	2.05*E* − 05	-1.3812	2.50*E* − 04	-0.7588	6.51*E* − 05	-0.5990	hsa_circ_0049547	-1.3519	1.43*E* − 04	hsa_circ_0069748	1.2636	1.42*E* − 04
DIAPH2	3.40*E* − 03	-0.5243	9.67*E* − 05	-1.4716	6.86*E* − 04	-0.5610	hsa_circ_0001901	-1.8577	1.66*E* − 04	hsa_circ_0002290	1.0164	1.44*E* − 04
TMEM47	5.69*E* − 05	-3.0122	1.27*E* − 05	0.8633	3.90*E* − 04	-1.4000	hsa_circ_0082139	-1.9232	1.77*E* − 04	hsa_circ_0083092	1.0164	1.44*E* − 04
HIGD1A	6.04*E* − 05	-1.0025	2.15*E* − 05	-0.7958	1.24*E* − 03	-0.8580	hsa_circ_0081968	-1.3107	1.91*E* − 04	hsa_circ_0006148	1.1531	1.87*E* − 04
TEAD3	6.72*E* − 05	-1.0911	2.43*E* − 04	1.0516	1.33*E* − 04	-0.6660	hsa_circ_0058934	-1.1454	2.01*E* − 04	hsa_circ_0008234	1.0489	1.90*E* − 04
FBXO32	7.28*E* − 05	-2.3381	3.50*E* − 02	-0.3892	1.30*E* − 02	-0.4000	hsa_circ_0077210	-1.1722	2.17*E* − 04	hsa_circ_0014405	1.0097	2.08*E* − 04
AHR	1.57*E* − 04	0.9134	5.22*E* − 09	1.4176	1.55*E* − 02	0.3310	hsa_circ_0045306	-0.8946	2.27*E* − 04	hsa_circ_0000860	0.6861	2.17*E* − 04
KIAA0226L	2.07*E* − 04	1.2992	3.40*E* − 06	-0.9575	4.38*E* − 04	0.6600	hsa_circ_0007720	-1.0737	2.62*E* − 04	hsa_circ_0005362	0.9872	2.38*E* − 04
PPP2R2B	3.34*E* − 04	-1.1755	7.22*E* − 06	0.7996	3.38*E* − 04	-0.9560	hsa_circ_0046882	-1.5741	2.67*E* − 04	hsa_circ_0003057	0.9963	2.63*E* − 04
RAB37	6.57*E* − 04	1.4399	1.78*E* − 04	0.5946	3.82*E* − 04	0.4000	hsa_circ_0084669	-1.4169	3.18*E* − 04	hsa_circ_0001727	1.1045	2.75*E* − 04
LDB3	8.46*E* − 04	-0.8926	1.94*E* − 04	1.0648	2.97*E* − 05	-0.6760	hsa_circ_0004299	-1.2148	3.38*E* − 04	hsa_circ_0078373	0.6885	2.99*E* − 04
LGR6	1.14*E* − 03	-1.0623	4.27*E* − 08	-1.5357	6.21*E* − 05	-0.6850	hsa_circ_0057691	-2.4049	4.09*E* − 04	hsa_circ_0002124	1.2467	3.02*E* − 04
CAV2	1.28*E* − 03	-1.5381	1.22*E* − 04	-1.1582	2.96*E* − 04	-0.6090	hsa_circ_0002168	-1.2299	4.11*E* − 04	hsa_circ_0091671	1.2030	3.05*E* − 04
AK4	1.71*E* − 03	-1.1842	2.38*E* − 04	-1.0428	2.54*E* − 06	-0.8010	hsa_circ_0085784	-0.6458	4.35*E* − 04	hsa_circ_0002988	1.0584	3.34*E* − 04
PDGFA	3.45*E* − 03	-1.5710	6.20*E* − 06	-0.7961	3.23*E* − 08	-0.8660	hsa_circ_0083182	-1.1488	4.39*E* − 04	hsa_circ_0004466	0.7994	3.43*E* − 04

**Table 2:** Differentially expressed miRNAs in different cells compared to AAAs.

Cell type	miRNA						
M1	hsa-let-7b^∗^	hsa-miR-1280	hsa-miR-149^∗^	hsa-miR-30e	hsa-miR-557		
M2	hsa-miR-1182	hsa-miR-127-3p	hsa-miR-1307	hsa-miR-132	hsa-miR-1321	hsa-miR-550a	hsa-miR-340^∗^
	hsa-miR-18b	hsa-miR-140-3p	hsa-miR-196b	hsa-miR-29c	hsa-miR-30e^∗^	hsa-miR-584	hsa-miR-342-5p
	hsa-miR-362-3p	hsa-miR-362-5p	hsa-miR-376a	hsa-miR-454	hsa-miR-486-5p	hsa-miR-610	hsa-miR-361-5p
	hsa-miR-1323	hsa-miR-136	hsa-miR-139-3p	hsa-miR-181a-2^∗^	hsa-miR-181a^∗^	hsa-miR-631	hsa-miR-663
	hsa-miR-328	hsa-miR-340					
SMC	hsa-let-7c	hsa-miR-29b	hsa-miR-145^∗^				
M1/M2	hsa-miR-660	hsa-miR-1207-5p	hsa-miR-144^∗^	hsa-miR-331-3p	hsa-miR-30c-1^∗^	hsa-miR-21^∗^	
M1/SMC	hsa-miR-17	hsa-miR-204	hsa-miR-23b	hsa-miR-28-5p	hsa-miR-187^∗^	hsa-miR-1224-5p	
M2/SMC	hsa-miR-150	hsa-miR-1825	hsa-miR-21	hsa-miR-210	hsa-miR-29c^∗^	hsa-miR-423-5p	hsa-miR-483-5p
M1/M2/SMC	hsa-let-7e	hsa-let-7f	hsa-miR-101	hsa-miR-107	hsa-miR-10a	hsa-miR-451	hsa-miR-193a-5p
	hsa-miR-1238	hsa-miR-1281	hsa-miR-1290	hsa-miR-146a	hsa-miR-181a	hsa-miR-497	hsa-miR-197
	hsa-miR-320a	hsa-miR-320b	hsa-miR-320d	hsa-miR-342-3p	hsa-miR-34a	hsa-miR-513a-5p	hsa-miR-199a-3p
	hsa-miR-296-5p	hsa-miR-29a	hsa-miR-30a	hsa-miR-574-5p	hsa-miR-877^∗^	hsa-miR-24	hsa-miR-36b
	hsa-miR-1181	hsa-miR-1183	hsa-miR-1226^∗^	hsa-miR-1228	hsa-miR-1234		

**Table 3:** Gene Ontology term enrichment and genome pathway enrichment analysis for differentially expressed circRNAs binding to mRNAs.

	Term	Count	*P* value	Genes
Gene_Ontology	GO:0050434~positive regulation of viral transcription	3	1.22*E* − 03	CTDP1, MDFIC, RSF1
	GO:0006027~glycosaminoglycan catabolic process	2	4.55*E* − 02	GPC1, IDS
	GO:0000922~spindle pole	3	1.56*E* − 02	CTDP1, CSPP1, CEP95
	GO:0005654~nucleoplasm	11	1.76*E* − 02	NFAT5, SCAF8, CTDP1, MDFIC, FIP1L1, SATB2, NCAPG2, MAPKAPK5, RSF1, PHC3, FOXP1
	GO:0005819~spindle	3	1.90*E* − 02	CTDP1, CSPP1, NUSAP1
	GO:0031519~PcG protein complex	2	4.64*E* − 02	UBAP2L, PHC3
	GO:0015629~actin cytoskeleton	3	5.59*E* − 02	CTDP1, ARPC1B, PHC3
	GO:0004721~phosphoprotein phosphatase activity	2	8.19*E* − 02	PPP4R1, CTDP1
KEGG_PATHWAY	Activation of Rac	1	1.39*E* − 02	GPC1
	TRAF6 mediated IRF7 activation in TLR7/8 or 9 signaling	1	1.39*E* − 02	TMEM189-UBE2V1
	Inactivation of Cdc42 and Rac	1	1.39*E* − 02	GPC1
	Role of Abl in Robo-Slit signaling	1	1.56*E* − 02	GPC1
	IRAK1 recruits the IKK complex upon TLR7/8 or 9 stimulation	1	1.74*E* − 02	TMEM189-UBE2V1
	IRAK1 recruits the IKK complex	1	1.74*E* − 02	TMEM189-UBE2V1
	Signaling by Robo receptor	1	3.95*E* − 02	GPC1
	Abortive elongation of HIV-1 transcript in the absence of Tat	1	3.95*E* − 02	CTDP1
	NOD1/2 signaling pathway	1	4.46*E* − 02	TMEM189-UBE2V1
	Beta2 integrin cell surface interactions	1	4.79*E* − 02	ITGAL

**Table 4:** Gene Ontology term enrichment and genome pathway enrichment analysis for differentially expressed mRNAs of the downregulation group.

	Term	Count	*P* value	Genes
Gene_Ontology	GO:0006936~muscle contraction	6	9.82*E* − 04	MYOM1, ITGB5, CALD1, FXYD1, SORBS1, MYLK
	GO:0007160~cell-matrix adhesion	5	3.83*E* − 03	ITGB5, ITGA10, ILK, SORBS1, FBLN5
	GO:1903779~regulation of cardiac conduction	4	7.32*E* − 03	PLN, TRPC1, FXYD1, ATP1A2
	GO:0030335~positive regulation of cell migration	6	1.00*E* − 02	MYOC, NTRK3, PDGFA, ILK, LGR6, MYLK
	GO:1901897~regulation of relaxation of cardiac muscle	2	2.11*E* − 02	PLN, CAMK2G
	GO:0051897~positive regulation of protein kinase B signaling	4	2.18*E* − 02	MYOC, PDGFA, ILK, AKR1C2
	GO:0015758~glucose transport	3	2.28*E* − 02	EDNRA, SORBS1, KLF15
	GO:0034446~substrate adhesion-dependent cell spreading	3	2.97*E* − 02	ILK, PARVA, FGFRL1
	GO:0070836~caveola assembly	2	3.49*E* − 02	CAV2, PACSIN2
	GO:0051260~protein homooligomerization	5	3.72*E* − 02	PLN, STOM, KCTD3, TERF1, FGFRL1
	GO:0043547~positive regulation of GTPase activity	9	4.77*E* − 02	NET1, BTC, ARHGEF12, TBXA2R, CAV2, PDGFA, ASAP2, DNM1L, CAMK2G
	GO:0045822~negative regulation of heart contraction	2	4.86*E* − 02	PLN, ATP1A2
	GO:0043235~receptor complex	7	2.43*E* − 04	GHR, ITGB5, TRPC1, NTRK3, ROR1, VLDLR, GPRC5C
	GO:0005783~endoplasmic reticulum	14	4.50*E* − 03	AOC3, CRTAP, MYOC, P3H3, TEX2, PLA2G16, PGRMC1, RCN2, PLN, CYB5R3, TMEM98, STOM, KIF1C, DNM1L
	GO:0005887~integral component of plasma membrane	19	7.89*E* − 03	JAG1, CAV2, PCDH7, TRPC1, NTRK3, GHR, BACE1, TRO, EDNRA, SYPL1, TBXA2R, FXYD1, AGTR1, STOM, TSPAN2, ROR1, LGR6, GPRC5C, PLPP1
	GO:0005901~caveola	4	1.01*E* − 02	CAV2, PRKCDBP, PACSIN2, ATP1A2
	GO:0005788~endoplasmic reticulum lumen	6	1.05*E* − 02	BACE1, CRTAP, RCN2, ESD, EOGT, PDGFA
	GO:0005925~focal adhesion	8	1.83*E* − 02	CSRP1, ITGB5, CAV2, PACSIN2, ILK, PARVA, SORBS1, AIF1L
	GO:0043234~protein complex	8	2.35*E* − 02	PLN, DTNA, CAV2, PRKCDBP, ILK, HIGD1A, DNM1L, AIF1L
	GO:0005515~protein binding	78	6.45*E* − 03	AVEN, MYOM1, SCOC, ITGB5, JADE1, ILK, VLDLR, MYLK, GHR, TRO, TRIM6, EDNRA, KIF1C, DNM1L, SKP1, UPF2, PIAS3, MBNL1, ARHGEF12, MYOC, TRPC1, CSNK2A2, BCKDHB, TERF1, KLF15, BTC, BACE1, PLA2G16, MORF4L1, INPP4B, RCN2, SYF2, NME7, AGTR1, ROR1, PPP1R12B, TAGLN, RTN1, FAM129A, PDGFA, ATP1A2, PDS5B, ASAP2, FBLN5, NCKIPSD, PGRMC1, PGRMC2, UGP2, PLN, TBXA2R, CALD1, ESD, MIER1, MXI1, PRKCDBP, PACSIN2, STOM, TSPAN2, RPRD1A, CAMK2G, MPZL2, AOC3, DTNA, JAG1, SSB, TMEM30B, NDUFA4, CAV2, NTRK3, PARVA, SORBS1, FBXO32, TMEM159, CCNG1, ANP32B, COPS8, RCOR3, LGR6
	GO:0008092~cytoskeletal protein binding	3	4.64*E* − 02	NCKIPSD, PACSIN2, SORBS1
	GO:0003779~actin binding	6	4.99*E* − 02	DIAPH2, TAGLN, CALD1, PARVA, SORBS1, MYLK
	GO:0042803~protein homodimerization activity	10	7.90*E* − 02	GHR, AOC3, MYOM1, CAV2, PDGFA, STOM, TERF1, DNM1L, CAMK2G, FBLN5
KEGG_Pathway	hsa04510: focal adhesion	8	1.17*E* − 03	ITGB5, CAV2, ITGA10, PDGFA, ILK, PARVA, PPP1R12B, MYLK
	hsa04270: vascular smooth muscle contraction	6	2.31*E* − 03	EDNRA, ARHGEF12, CALD1, AGTR1, PPP1R12B, MYLK
	hsa04810: regulation of actin cytoskeleton	7	6.28*E* − 03	DIAPH2, ARHGEF12, ITGB5, ITGA10, PDGFA, PPP1R12B, MYLK
	hsa04020: calcium signaling pathway	6	1.37*E* − 02	EDNRA, PLN, TBXA2R, AGTR1, CAMK2G, MYLK
	hsa04022: cGMP-PKG signaling pathway	5	3.70*E* − 02	EDNRA, PLN, AGTR1, ATP1A2, MYLK

**Table 5:** Gene Ontology term enrichment and genome pathway enrichment analysis for differentially expressed mRNAs of the ceRNA network.

	Term	Count	*P* value	Genes
Gene_Ontology	GO:0008285~negative regulation of cell proliferation	3	4.19*E* − 02	COPS8, SOD2, FGFRL1
	GO:0006357~regulation of transcription from RNA polymerase II promoter	3	5.08*E* − 02	KLF9, AHR, SOD2
	GO:0001889~liver development	2	6.00*E* − 02	AK4, SOD2
	GO:0008180~COP9 signalosome	2	2.84*E* − 02	NCKIPSD, COPS8
	GO:0030133~transport vesicle	2	7.54*E* − 02	PLEKHF2, FGFRL1
	GO:0003677~DNA binding	5	5.41*E* − 02	NFIC, MIER1, KLF9, AHR, SOD2
KEGG_PATHWAY	bta04146: peroxisome	1	4.38*E* − 02	SOD2
	bta04068: FoxO signaling pathway	1	6.81*E* − 02	SOD2

## Data Availability

The GEO dataset data used to support the findings of this study are available from the corresponding author upon request.

## Supplementary Materials

Supplementary Materials Supplementary Figure 1: box plot of each sample in the four datasets. The blue line in the box represents the median, and circles represent outliers: (A) GSE144431; (B) GSE47472; (C) GSE7084; (D) GSE57691. Supplementary Table 1: common DEGs of different regulation methods in the three datasets. Supplementary Table 2: prediction of partial miRNAs binding to circRNAs. Supplementary Table 3: Gene Ontology term enrichment and genome pathway enrichment analysis for differentially expressed mRNAs of the upregulation group.
