# Supplementary material for: Construction of the circRNA-miRNA-mRNA Regulatory Network of an Abdominal Aortic Aneurysm to Explore Its Potential Pathogenesis
Source: Dis Markers. 2021 Nov 5;2021:9916881. doi: 10.1155/2021/9916881 (PMC8589483; doi:10.1155/2021/9916881)
Supplement: Supplementary Materials — Supplementary Figure 1: box plot of each sample in the four datasets. The blue line in the box represents the median, and circles represent outliers: (A) GSE144431; (B) GSE47472; (C) GSE7084; (D) GSE57691. Supplementary Table 1: common DEGs of different regulation methods in the three datasets. Supplementary Table 2: prediction of partial miRNAs binding to circRNAs. Supplementary Table 3: Gene Ontology term enrichment and genome pathway enrichment analysis for differentially expressed mRNAs of the upregulation group. [file 9916881.f1.docx]

# Supplementary Material

## Supplementary Figures


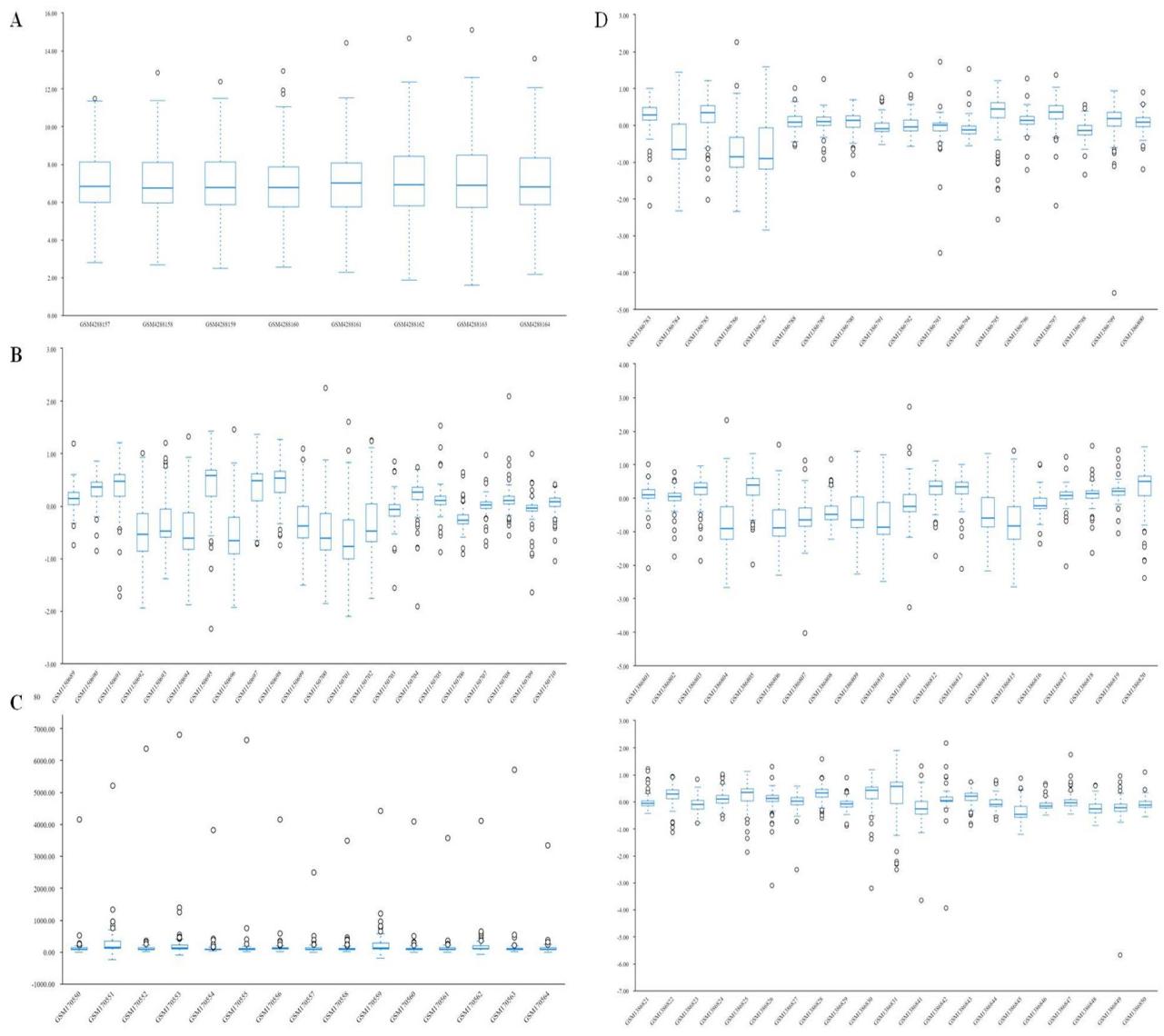


**Supplementary Figure 1.** Box plot of each sample in the four datasets. The blue line in the box represents the median, circles represent outliers;(A) GSE144431;(B) GSE47472;(C) GSE7084;(D) GSE57691.

## Supplementary Table

**Supplementary Table 1.** Common DEGs of different regulation methods in three datasets.

|  | GSE7084 | | | GSE47472 | | | GSE57691 | | |
| --- | --- | --- | --- | --- | --- | --- | --- | --- | --- |
| Gene.symbol | adj.P.Val | P.Value | logFC | adj.P.Val | P.Value | logFC | adj.P.Val | P.Value | logFC |
| ANTXR1 | 4.65E-02 | 7.59E-04 | -2.2475 | 2.19E-01 | 3.82E-02 | 0.2420 | 1.61E-01 | 2.47E-02 | -0.7060 |
| CCNB1IP1 | 9.09E-02 | 2.08E-03 | -1.4135 | 2.21E-01 | 3.87E-02 | 0.2390 | 5.78E-04 | 5.30E-06 | -0.5410 |
| ANKRD6 | 1.24E-01 | 3.32E-03 | -0.7161 | 2.39E-04 | 3.94E-06 | -0.9490 | 6.69E-02 | 7.29E-03 | 0.8259 |
| RGN | 1.40E-01 | 3.96E-03 | -0.9504 | 1.97E-02 | 1.43E-03 | -1.0635 | 4.85E-02 | 4.77E-03 | 0.7834 |
| PPP2R3A | 1.49E-01 | 4.35E-03 | -0.5847 | 9.61E-02 | 1.19E-02 | 0.4030 | 1.41E-03 | 4.37E-05 | 0.9113 |
| LRRC1 | 1.56E-01 | 4.62E-03 | -0.9110 | 1.62E-01 | 2.48E-02 | 0.2690 | 0.000727 | 7.43E-06 | -0.7610 |
| TSC22D1 | 3.29E-01 | 1.45E-02 | -1.4107 | 1.74E-01 | 2.76E-02 | 0.2860 | 6.37E-02 | 6.61E-03 | -0.5690 |
| PNRC2 | 3.37E-01 | 1.50E-02 | -0.5798 | 6.64E-02 | 7.04E-03 | 0.2800 | 1.26E-01 | 1.74E-02 | -0.6400 |
| CDK5RAP2 | 3.49E-01 | 1.60E-02 | -0.9198 | 1.79E-02 | 1.03E-03 | 0.4940 | 9.24E-02 | 1.13E-02 | -0.5270 |
| MATR3 | 3.62E-01 | 1.69E-02 | -0.5529 | 5.21E-02 | 4.89E-03 | 0.4000 | 1.55E-01 | 2.33E-02 | -0.6590 |
| ZNF627 | 3.89E-01 | 1.91E-02 | -0.5924 | 1.52E-01 | 2.28E-02 | 0.2190 | 2.13E-03 | 3.89E-05 | -0.8150 |
| DMD | 4.01E-01 | 1.99E-02 | -0.6172 | 5.11E-06 | 2.34E-08 | -1.2989 | 1.09E-04 | 1.34E-06 | 1.0479 |
| NAPEPLD | 4.50E-01 | 2.39E-02 | -0.5363 | 5.82E-02 | 5.75E-03 | 0.3040 | 2.08E-01 | 3.55E-02 | -0.2830 |
| PAIP1 | 5.02E-01 | 2.83E-02 | -0.7298 | 1.77E-01 | 2.82E-02 | 0.2380 | 5.60E-03 | 2.72E-04 | 1.0303 |
| ARPP21 | 6.18E-01 | 3.91E-02 | -0.2266 | 6.40E-06 | 3.12E-08 | -1.3143 | 8.45E-02 | 1.00E-02 | 0.4690 |
| LTBP4 | 7.08E-01 | 4.92E-02 | -1.3514 | 1.99E-01 | 3.34E-02 | 0.3000 | 1.60E-01 | 2.34E-02 | 0.3285 |
| LILRB2 | 8.18E-04 | 1.02E-06 | 2.1184 | 2.87E-02 | 2.39E-03 | -0.5046 | 1.90E-02 | 1.36E-03 | 1.1100 |
| ITGB1BP1 | 4.11E-03 | 1.97E-05 | 1.1488 | 8.16E-06 | 4.43E-08 | -1.1687 | 4.74E-02 | 4.27E-03 | -0.5350 |
| USP14 | 1.24E-02 | 1.08E-04 | 0.9438 | 8.24E-02 | 9.62E-03 | 0.3140 | 2.04E-02 | 1.25E-03 | -0.5900 |
| TCF7 | 1.89E-02 | 1.96E-04 | 2.3496 | 2.77E-02 | 2.28E-03 | -0.5046 | 3.50E-02 | 2.77E-03 | -0.3280 |
| COQ2 | 2.25E-02 | 2.48E-04 | 1.1219 | 1.69E-01 | 2.51E-02 | -0.7460 | 2.18E-01 | 3.81E-02 | -0.3650 |
| ZNF667 | 3.04E-02 | 3.91E-04 | 0.7506 | 1.50E-02 | 9.78E-04 | -0.6570 | 8.11E-02 | 9.45E-03 | 0.4323 |
| SPN | 3.44E-02 | 4.77E-04 | 0.9784 | 2.71E-02 | 2.22E-03 | -0.5512 | 2.14E-02 | 1.35E-03 | -0.8510 |
| PRR13 | 1.07E-01 | 2.65E-03 | 0.8128 | 2.26E-01 | 4.02E-02 | 0.2910 | 2.54E-01 | 4.77E-02 | -0.3460 |
| ST8SIA4 | 1.18E-01 | 3.08E-03 | 0.5499 | 6.43E-02 | 6.91E-03 | -0.5938 | 2.38E-01 | 4.03E-02 | 0.6633 |
| IFIT3 | 1.41E-01 | 4.00E-03 | 0.8066 | 3.61E-06 | 1.42E-08 | -1.3281 | 1.27E-01 | 1.77E-02 | --0.5100 |
| UBE2D2 | 2.36E-01 | 8.69E-03 | 0.4155 | 1.80E-01 | 2.73E-02 | -0.3274 | 4.56E-03 | 1.28E-04 | -0.3790 |
| TPST2 | 2.46E-01 | 9.31E-03 | 0.8317 | 1.33E-01 | 1.83E-02 | -0.4212 | 2.42E-01 | 4.42E-02 | -0.4710 |
| MTSS1 | 2.51E-01 | 9.60E-03 | 0.7092 | 2.29E-01 | 4.10E-02 | 0.3080 | 3.64E-02 | 2.93E-03 | -0.7890 |
| FAAP100 | 2.62E-01 | 1.03E-02 | 0.8654 | 5.87E-02 | 6.13E-03 | -0.3976 | 4.22E-02 | 3.63E-03 | -0.4870 |
| TFIP11 | 2.87E-01 | 1.17E-02 | 0.7807 | 2.82E-03 | 6.07E-05 | 0.4280 | 2.04E-01 | 3.46E-02 | -0.4630 |
| TADA3 | 3.13E-01 | 1.33E-02 | 0.6968 | 2.23E-02 | 1.70E-03 | -0.7753 | 7.92E-02 | 9.09E-03 | -0.5140 |
| GLB1 | 3.56E-01 | 1.65E-02 | 0.7144 | 1.15E-01 | 1.50E-02 | -0.3663 | 1.01E-01 | 1.27E-02 | -0.5640 |
| CCM2 | 3.65E-01 | 1.72E-02 | 0.6531 | 2.00E-03 | 6.98E-05 | -0.9534 | 3.76E-02 | 3.08E-03 | -0.4760 |
| ARSB | 3.72E-01 | 1.76E-02 | 0.3459 | 1.07E-04 | 1.29E-06 | -1.1369 | 9.11E-03 | 3.66E-04 | -0.6900 |
| C11orf1 | 3.82E-01 | 1.85E-02 | 0.5390 | 5.34E-03 | 2.57E-04 | -0.8122 | 2.94E-03 | 6.50E-05 | -0.5600 |
| CD58 | 3.90E-01 | 1.92E-02 | 0.5545 | 5.15E-03 | 2.44E-04 | -0.6579 | 4.58E-02 | 4.07E-03 | -0.5920 |
| GTDC1 | 4.88E-01 | 2.70E-02 | 0.2147 | 1.48E-01 | 2.19E-02 | 0.2710 | 1.65E-02 | 9.11E-04 | -0.3240 |
| NUBP2 | 4.92E-01 | 2.74E-02 | 0.5181 | 2.61E-02 | 2.11E-03 | -0.6326 | 1.70E-01 | 2.68E-02 | -0.3020 |
| TIMP2 | 5.01E-01 | 2.81E-02 | 0.8312 | 2.37E-01 | 4.00E-02 | -0.5832 | 3.38E-02 | 2.63E-03 | -0.7730 |
| GON7 | 5.02E-01 | 2.84E-02 | 0.3639 | 2.34E-01 | 3.92E-02 | -0.4121 | 1.86E-02 | 1.09E-03 | -0.4990 |
| CAMSAP1 | 5.46E-01 | 3.22E-02 | 0.4753 | 7.78E-03 | 4.22E-04 | -0.6454 | 2.05E-02 | 1.26E-03 | -0.4660 |
| VPS41 | 5.82E-01 | 3.57E-02 | 0.3494 | 4.09E-02 | 3.46E-03 | 0.2900 | 8.42E-02 | 9.87E-03 | -0.5640 |
| CLEC2D | 5.89E-01 | 3.63E-02 | 0.7069 | 4.59E-02 | 4.08E-03 | 0.4550 | 2.99E-02 | 2.21E-03 | -0.7800 |
| STARD8 | 6.24E-01 | 3.97E-02 | 0.6417 | 2.50E-01 | 4.31E-02 | -0.3968 | 4.07E-03 | 1.08E-04 | -0.7250 |
| CCNYL1 | 6.28E-01 | 4.02E-02 | 0.4336 | 6.03E-02 | 6.08E-03 | 0.2680 | 9.10E-02 | 1.10E-02 | -0.2260 |
| GRAP | 6.33E-01 | 4.07E-02 | 0.3197 | 1.14E-03 | 3.25E-05 | -0.8132 | 1.81E-01 | 2.76E-02 | 0.3200 |

# Supplementary Table 2. Prediction of partial miRNAs binding to circRNAs.

| circRNA | | miRNA | | | | |
| --- | --- | --- | --- | --- | --- | --- |
| Down-regulation | hsa_circ_0005073 | hsa-miR-3662 | hsa-miR-30a-3p | hsa-miR-30d-3p | hsa-miR-30e-3p | hsa-miR-548ac |
|  | hsa_circ_0006845 | hsa-miR-6842-3p | hsa-miR-106a-3p | hsa-miR-198 | hsa-miR-3064-5p | hsa-miR-382-5p |
|  | hsa_circ_0044885 | hsa-miR-548c-3p | hsa-miR-153-5p | hsa-miR-3671 | hsa-miR-3926 | hsa-miR-4668-3p |
|  | hsa_circ_0011449 | hsa-miR-4459 | hsa-miR-4695-5p | hsa-miR-4719 | hsa-miR-4779 | hsa-miR-4668-3p |
|  | hsa_circ_0082140 | hsa-miR-4505 | hsa-miR-3191-5p | hsa-miR-3663-5p | hsa-miR-4474-3p | hsa-miR-4757-5p |
|  | hsa_circ_0049547 | hsa-miR-134-3p | hsa-let-7c-3p | hsa-miR-1243 | hsa-miR-224-3p | hsa-miR-3153 |
|  | hsa_circ_0001901 | hsa-miR-1208 | hsa-miR-6760-3p | hsa-miR-134-3p | hsa-miR-146b-3p | hsa-miR-1910-3p |
|  | hsa_circ_0082139 | hsa-miR-4505 | hsa-miR-6842-3p | hsa-miR-1248 | hsa-miR-130a-5p | hsa-miR-1587 |
|  | hsa_circ_0081968 | hsa-miR-3121-5p | hsa-miR-3662 | hsa-miR-1184 | hsa-miR-1205 | hsa-miR-17-3p |
|  | hsa_circ_0058934 | hsa-miR-146b-3p | hsa-miR-149-3p | hsa-miR-4728-5p | hsa-miR-6785-5p | hsa-miR-6799-3p |
|  | hsa_circ_0077210 | hsa-miR-25-3p | hsa-miR-32-5p | hsa-miR-4753-3p | hsa-miR-548aj-5p | hsa-miR-548g-5p |
|  | hsa_circ_0045306 | hsa-miR-1293 | hsa-miR-2467-5p | hsa-miR-3691-5p | hsa-miR-384 | hsa-miR-3975 |
|  | hsa_circ_0007720 | hsa-miR-125a-3p | hsa-miR-1269a | hsa-miR-1269b | hsa-miR-320e | hsa-miR-573 |
|  | hsa_circ_0046882 | hsa-miR-4678 | hsa-miR-4686 | hsa-miR-4762-3p | hsa-miR-6736-3p | hsa-miR-1-5p |
|  | hsa_circ_0084669 | hsa-miR-4753-3p | hsa-miR-1299 | hsa-miR-1343-3p | hsa-miR-193a-5p | hsa-miR-3612 |
|  | hsa_circ_0004299 | hsa-miR-6815-3p | hsa-miR-6886-5p | hsa-miR-103a-2-5p | hsa-miR-1224-5p | hsa-miR-1270 |
|  | hsa_circ_0057691 | hsa-miR-1273g-3p | hsa-miR-185-5p | hsa-miR-6731-5p | hsa-miR-6764-3p | hsa-miR-8085 |
|  | hsa_circ_0002168 | hsa-miR-15a-3p | hsa-miR-1910-3p | hsa-miR-1911-3p | hsa-miR-298 | hsa-miR-3158-3p |
|  | hsa_circ_0085784 | hsa_circ_0085784 | hsa_circ_0085784 | hsa_circ_0085784 | hsa_circ_0085784 | hsa_circ_0085784 |
|  | hsa_circ_0083182 | hsa-miR-126-5p | hsa-miR-532-5p | hsa-miR-20a-3p | hsa-miR-3190-3p | hsa-miR-383-5p |
| Up-regulation | hsa_circ_0037128 | hsa-miR-1207-5p | hsa-miR-3619-5p | hsa-miR-4736 | hsa-miR-4763-3p | hsa-miR-4781-5p |
|  | hsa_circ_0017972 | hsa-miR-1265 | hsa-miR-1914-3p | hsa-miR-2861 | hsa-miR-3192-5p | hsa-miR-4456 |
|  | hsa_circ_0039557 | hsa-miR-19a-3p | hsa-miR-19b-3p | hsa-miR-3942-3p | hsa-miR-548aw | hsa-miR-583 |
|  | hsa_circ_0062011 | hsa-miR-6865-5p | hsa-miR-1205 | hsa-miR-1247-5p | hsa-miR-1286 | hsa-miR-150-3p |
|  | hsa_circ_0000690 | hsa-miR-1272 | hsa-miR-6736-3p | hsa-miR-7161-3p | hsa-miR-9-5p | hsa-miR-1184 |
|  | hsa_circ_0069748 | hsa-miR-6134 | hsa-miR-6880-5p | hsa-miR-3190-3p | hsa-miR-6851-5p | hsa-miR-765 |
|  | hsa_circ_0002290 | hsa-miR-34b-5p | hsa-miR-3714 | hsa-miR-449c-5p | hsa-miR-4659a-3p | hsa-miR-4659b-3p |
|  | hsa_circ_0083092 | hsa-miR-452-3p | hsa-miR-1236-3p | hsa-miR-4273 | hsa-miR-4677-5p | hsa-miR-512-5p |
|  | hsa_circ_0006148 | hsa-miR-3150b-3p | hsa-miR-4704-3p | hsa-miR-4784 | hsa-miR-5011-5p | hsa-let-7a-3p |
|  | hsa_circ_0008234 | hsa-miR-3692-5p | hsa-miR-93-3p | hsa-miR-1267 | hsa-miR-367-5p | hsa-miR-127-5p |
|  | hsa_circ_0014405 | hsa-miR-5093 | hsa-miR-2276-3p | hsa-miR-296-3p | hsa-miR-3190-5p | hsa-miR-4435 |
|  | hsa_circ_0000860 | hsa-miR-3692-5p | hsa-miR-7153-3p | hsa-miR-767-5p | hsa-miR-1226-3p | hsa-miR-1236-3p |
|  | hsa_circ_0005362 | hsa-miR-3692-5p | hsa-miR-558 | hsa-miR-30c-1-3p | hsa-miR-4731-5p | hsa-miR-653-5p |
|  | hsa_circ_0003057 | hsa-miR-4474-3p | hsa-miR-7108-5p | hsa-miR-1255a | hsa-miR-1255b-5p | hsa-miR-134-5p |
|  | hsa_circ_0001727 | hsa-miR-3661 | hsa-miR-203b-3p | hsa-miR-3189-3p | hsa-miR-3192-5p | hsa-miR-4691-3p |
|  | hsa_circ_0078373 | hsa-miR-4659a-5p | hsa-miR-4659b-5p | hsa-miR-512-3p | hsa-miR-6817-5p | hsa-miR-106a-5p |
|  | hsa_circ_0002124 | hsa-miR-29b-2-5p | hsa-miR-4760-5p | hsa-miR-1267 | hsa-miR-1273f | hsa-miR-29b-1-5p |
|  | hsa_circ_0091671 | hsa-miR-18a-5p | hsa-miR-330-3p | hsa-miR-335-3p | hsa-miR-425-3p | hsa-miR-4476 |
|  | hsa_circ_0002988 | hsa-miR-1254 | hsa-miR-1287-5p | hsa-miR-3116 | hsa-miR-3690 | hsa-miR-4638-3p |
|  | hsa_circ_0004466 | hsa-miR-342-5p | hsa-miR-422a | hsa-miR-4638-3p | hsa-miR-224-5p | hsa-miR-3064-5p |

# Supplementary Table 3. Gene ontology term enrichment and genomes pathway enrichment analysis for differentially expressed mRNAs of up-regulation group.

|  | Term | Count | PValue | Genes |
| --- | --- | --- | --- | --- |
| Gene_Ontology | GO:0050852~T cell receptor signaling pathway | 5 | 2.19E-04 | ITK, PDE4B, PTPN22, CD3D, PAG1 |
|  | GO:0032496~response to lipopolysaccharide | 5 | 3.24E-04 | CD96, CCR7, PTPN22, SOD2, PTGS2 |
|  | GO:0090023~positive regulation of neutrophil chemotaxis | 3 | 8.96E-04 | CXCL8, CAMK1D, CCR7 |
|  | GO:0006954~inflammatory response | 5 | 6.94E-03 | GBP5, CXCL8, CAMK1D, CCR7, PTGS2 |
|  | GO:0001525~angiogenesis | 4 | 1.02E-02 | CXCL8, ANGPTL6, VASH1, PTGS2 |
|  | GO:0010042~response to manganese ion | 2 | 1.21E-02 | SOD2, PTGS2 |
|  | GO:0050868~negative regulation of T cell activation | 2 | 2.21E-02 | PTPN22, PAG1 |
|  | GO:0006935~chemotaxis | 3 | 2.52E-02 | CMTM7, CXCL8, CCR7 |
|  | GO:0002230~positive regulation of defense response to virus by host | 2 | 4.36E-02 | PTPN22, IL12RB1 |
|  | GO:0008285~negative regulation of cell proliferation | 4 | 4.54E-02 | CXCL8, RASSF5, SOD2, PTGS2 |
|  | GO:0035556~intracellular signal transduction | 4 | 4.74E-02 | ITK, CXCL8, RASSF5, PAG1 |
|  | GO:0006955~immune response | 4 | 5.28E-02 | CD96, IL1RN, CXCL8, CCR7 |
|  | GO:0005622~intracellular | 9 | 3.17E-03 | ITK, CSTA, IL1RN, CXCL8, RAB37, RASSF5, IL2RB, CCR7, PAG1 |
|  | GO:0046872~metal ion binding | 9 | 3.55E-02 | PLEKHF2, ITK, SLX1B, COL11A1, RASSF5, PDE4B, ATP2B1, SOD2, PTGS2 |
| KEGG_PATHWAY | hsa04060:Cytokine-cytokine receptor interaction | 5 | 1.98E-03 | CXCL8, IL2RB, IL21R, CCR7, IL12RB1 |
|  | hsa04630:Jak-STAT signaling pathway | 3 | 4.36E-02 | IL2RB, IL21R, IL12RB1 |
|  | hsa04062:Chemokine signaling pathway | 3 | 6.81E-02 | ITK, CXCL8, CCR7 |
